# A generalised approach for high-throughput instance segmentation of stomata in microscope images

**DOI:** 10.1186/s13007-021-00727-4

**Published:** 2021-03-09

**Authors:** Hiranya Jayakody, Paul Petrie, Hugo Jan de Boer, Mark Whitty

**Affiliations:** 1 School of Mechanical and Manufacturing Engineering, UNSW, Sydney, Australia; 2 South Australian Research and Development Institute, Urrbrae, Australia; 3 Department of Environmental Sciences, Copernicus institute of sustainable development, Utrecht University, Utrecht, Netherlands

**Keywords:** Automatic stomata detection, Microscope imagery, Mask R-CNN, Instance segmentation, High-throughput analysis, Machine learning

## Abstract

**Background:**

Stomata analysis using microscope imagery provides important insight into plant physiology, health and the surrounding environmental conditions. Plant scientists are now able to conduct automated high-throughput analysis of stomata in microscope data, however, existing detection methods are sensitive to the appearance of stomata in the training images, thereby limiting general applicability. In addition, existing methods only generate bounding-boxes around detected stomata, which require users to implement additional image processing steps to study stomata morphology. In this paper, we develop a fully automated, robust stomata detection algorithm which can also identify individual stomata boundaries regardless of the plant species, sample collection method, imaging technique and magnification level.

**Results:**

The proposed solution consists of three stages. First, the input image is pre-processed to remove any colour space biases occurring from different sample collection and imaging techniques. Then, a Mask R-CNN is applied to estimate individual stomata boundaries. The feature pyramid network embedded in the Mask R-CNN is utilised to identify stomata at different scales. Finally, a statistical filter is implemented at the Mask R-CNN output to reduce the number of false positive generated by the network. The algorithm was tested using 16 datasets from 12 sources, containing over 60,000 stomata. For the first time in this domain, the proposed solution was tested against 7 microscope datasets never seen by the algorithm to show the generalisability of the solution. Results indicated that the proposed approach can detect stomata with a precision, recall, and F-score of 95.10%, 83.34%, and 88.61%, respectively. A separate test conducted by comparing estimated stomata boundary values with manually measured data showed that the proposed method has an IoU score of 0.70; a 7% improvement over the bounding-box approach.

**Conclusions:**

The proposed method shows robust performance across multiple microscope image datasets of different quality and scale. This generalised stomata detection algorithm allows plant scientists to conduct stomata analysis whilst eliminating the need to re-label and re-train for each new dataset. The open-source code shared with this project can be directly deployed in Google Colab or any other Tensorflow environment.

## Background

Stomata are microscopic pores in the leaf surface that play a critical role in controlling photosynthesis and transpiration [1–3]. The apertures of these microscopic pores are controlled by two guard cells that surround each pore. The opening and closing of stomatal pores directly impact both CO$_{2}$ intake and water transpiration rate of a plant [4–6]. Hence, plant scientists study stomata behaviour to learn more about plant water stress as well as surrounding environmental changes [7–9]. In addition to studying living plants, scientists also use plant fossil cuticles to uncover climate change patterns by analysing stomatal density, size and behaviour [10–12].

Stomatal traits can be measured using both direct and indirect methods. The direct method involves stomata phenotyping using microscope images, whereas the indirect methods use porometers or infrared gas analysers to measure stomatal conductance and gas exchange to infer information on the aperture of the stomatal pores [8, 13, 14]. Among the two methods, stomata analysis through microscope images provides additional information such as stomata size, shape, orientation, density and patchiness [1, 6, 15–17]. However, microscope image analysis requires scientists to count and measure a large number of stomata in order to find statistically significant patterns, and this proves to be time consuming and cumbersome work if done manually. Software solutions such as ImageJ^®^ aim to automate this process to some extent, but such software require experts to either manually mark certain features of cells or tune a set of parameters before measurements can take place [18–20]. Such time-consuming steps force scientists to conduct their studies with fewer data points; thus the true potential of the dataset is never achieved.

Automatic detection and measurement of stomata have the potential to solve this problem. Reliable automation leads to high-throughput analysis, which allows researchers to conduct their work using all available data. Recent advancements in computer vision and machine learning have provided some promising solutions to achieve high-throughput stomata analysis.

Initial attempts to automatically detect stomata in microscope images involved classical image processing approaches. After Osama and Onoe’s initial attempt in 1985, many researchers implemented different types of traditional image morphology operations to achieve this goal [21–24]. Sole reliance on image morphological operations performs well when the background is featureless, and stomata are clearly visible on the image. However, this is not the case for many microscope datasets. More recently, sophisticated methods such as template matching, maximum stable external region extraction and wavelet spot detection were utilized to identify stomata [25–27]. These methods require images to be relatively in focus to operate reliably. However, image quality for microscope images can vary dramatically depending on data collection and imaging techniques.

Recent developments in machine learning and Convolutional Neural Networks (CNN) have opened up new avenues for rapid detection and measurement of stomata in microscope images. Research by Vialet-Chabrand and Brendel, and Jayakody et al. utilised Cascade Object Detection (COD) algorithms with Histogram of Oriented Gradients (HOG) and Haar-like features to detect stomata [17, 28, 29]. Simple machine learning techniques like COD require a large amount of training data and have proven to be less robust than the more sophisticated CNN based machine learning algorithms developed recently. With the introduction of transfer learning, researchers were able to re-train existing general CNN models for specific applications using small amount of data [30]. In a research area where data collection and ground truth generation are time consuming exercises, researchers quickly proceeded to adopt these novel CNN models. Toda et al. [31] used HOG features to find areas which contain stomata, and used a CNN to classify the state (open, partially open, closed) of Dayflower (Commelina communis) stomata, using a sliding window technique. Using a sliding window makes the algorithm computationally more expensive and requires additional parameters, especially if stomata of different sizes need to be detected. Sakoda et al. developed a framework to analyse the genetic diversity in stomatal density of Soybeans (Glycine max) using a Single Shot Multi-box Detector (SSD) [32]. The SSD approach eliminates the need for a sliding window, improving the speed of stomata detection [33]. This popular approach was also adopted by Bhugra et al. to detect and measure pores of different rice cultivars [15]. However, in all these machine learning based approaches, the researchers have focused on implementing the algorithm targeting a specific plant species and a uniform sample preparation and imaging approach, where stomata have limited variation in size and appearance. This means, despite these CNN networks showing promising results, significant changes are required in order for them to be adopted for new datasets. Therefore, implementing a generalised methodology which can detect stomata across a variety of plant species is critical to facilitating wide-spread and rapid stomata analysis.

A couple of recent works focused on building generalised stomata detection platforms. Casado and Heras introduced a stomata detection pipeline using a YOLO object detector [34, 35]. In addition to applying the YOLO detector to identify stomata on cotton, peanut and maize plants, they implemented a general machine learning pipeline using a Jupyter^®^ notebook environment where researchers can prepare, train and apply the YOLO algorithm to their microscope data. The solution still requires new users to carry out some implementation work, but saves time by providing all the necessary tools and processes required for stomata identification. Fetter et al. tackled the problem of generalising stomata detection by implementing the web tool “StomataCounter” [36]. Here, the authors use thousands of samples across multiple plant types imaged using different techniques and scale to implement a generalised stomata detector. A popular CNN classifier named AlexNet is combined with a sliding window to achieve the stomata detection goal. Although StomataCounter performs well across multiple plant types, the algorithm is not robust against scale invariance as the scale of the stomata depends on the sliding window size. Thus, prior knowledge of stomatal size is required for robust operation.

All CNN methods discussed above generate rectangular bounding-boxes around detected stomata. This is useful in counting the number of stomata, but if the user intends to investigate the morphological features of the stomata, additional image processing steps are required. These image processing steps cannot be easily adopted to new datasets without serious modifications and pre-processing.

Regardless of the image processing technique used for stomatal detection and measurement, all current approaches suffer from the following limitations:

1. None of the methods have the ability to directly measure the stomata boundary during the detection step. Instead, a bounding box surrounding the stomata is first detected. Additional morphological operations are required to determine the boundary of the stomata.
2. None of the methods perform well across stomata at different scales.
3. All methods, except for [36], focus on applying their method on a specific plant type using a specific data collection procedure. Thus, the performance of the algorithm is drastically reduced when applied to a new dataset.

**Fig. 1:**
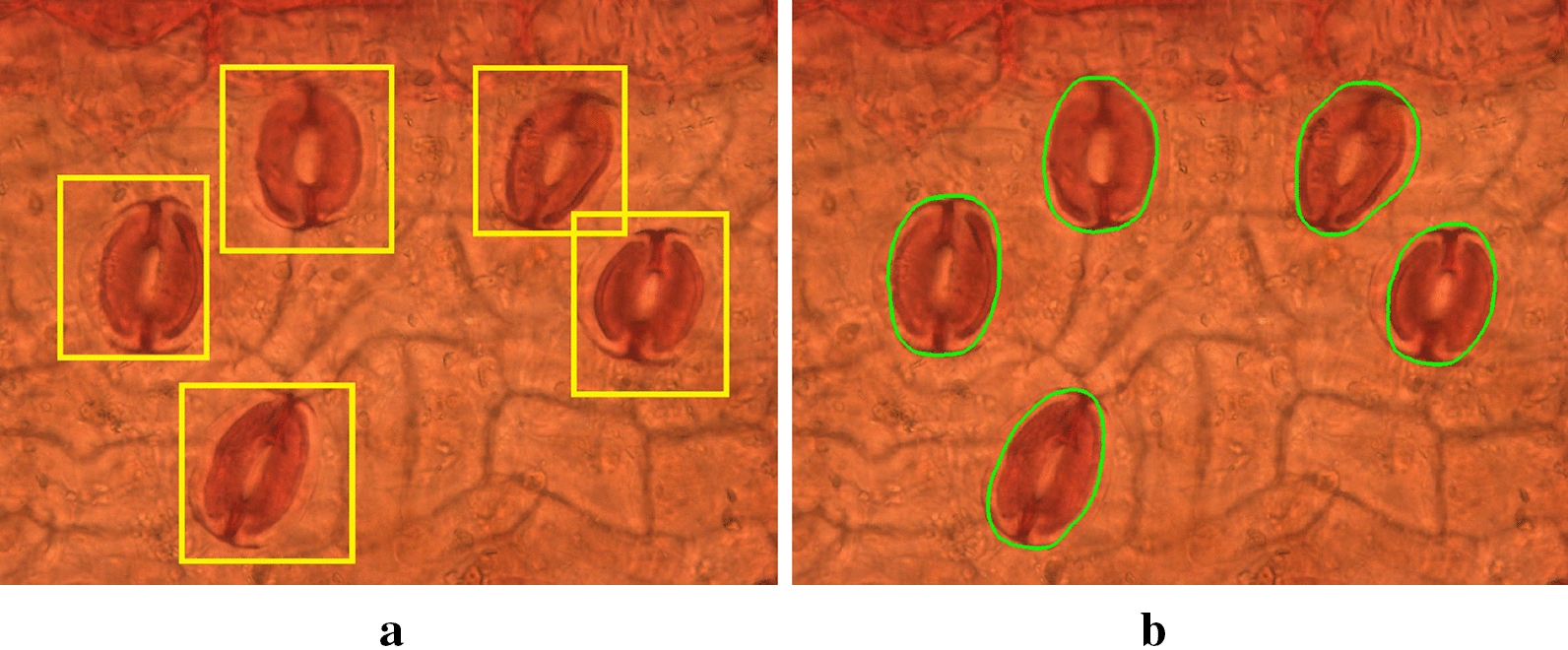
**a** Current state-of-the-art. **b** Instance segmentation of proposed method. Proposed method provides further insight into the morphological properties of stomata.

In this paper, we propose a robust framework to automatically detect stomata and directly measure stomatal area, which performs well across multiple plant species and is robust to different image magnification levels and image qualities. The proposed methodology utilizes the Mask R-CNN instance segmentation technique followed by a statistical filter to achieve this goal [37]. With this combination, our proposed method can:

1. directly determine the stomata boundary around the guard cell pair as shown in Fig. 1 instead of generating simple bounding boxes.
2. detect stomata at different scales utilizing the Feature Pyramid Network (FPN) implemented within the Mask R-CNN algorithm [38].
3. perform well across different plant species and sample preparation methods without any modification to the network.

Measuring the stomata boundary using instance segmentation allows researchers to directly determine stomata area, orientation and axis lengths. We also introduce a statistical filter at the Mask R-CNN model output to increase the overall precision of the algorithm when processing low quality images.

The proposed method was successfully tested using over 2800 microscope images containing more than 60,000 stomata. The overall accuracy, precision and F-scores of the algorithm were measured to be 95.10%, 83.34% and 88.61% respectively. For the first time in this domain, we successfully tested our algorithm against 7 microscope image datasets fully unknown to the neural network. A detailed breakdown of the results can be found in section: Results.

In situations where researchers do require increased accuracy, especially when the data is of poor quality, we provide comprehensive instructions and code to re-train our algorithm using less than 15 training images.

## Methods

**Fig. 2:**
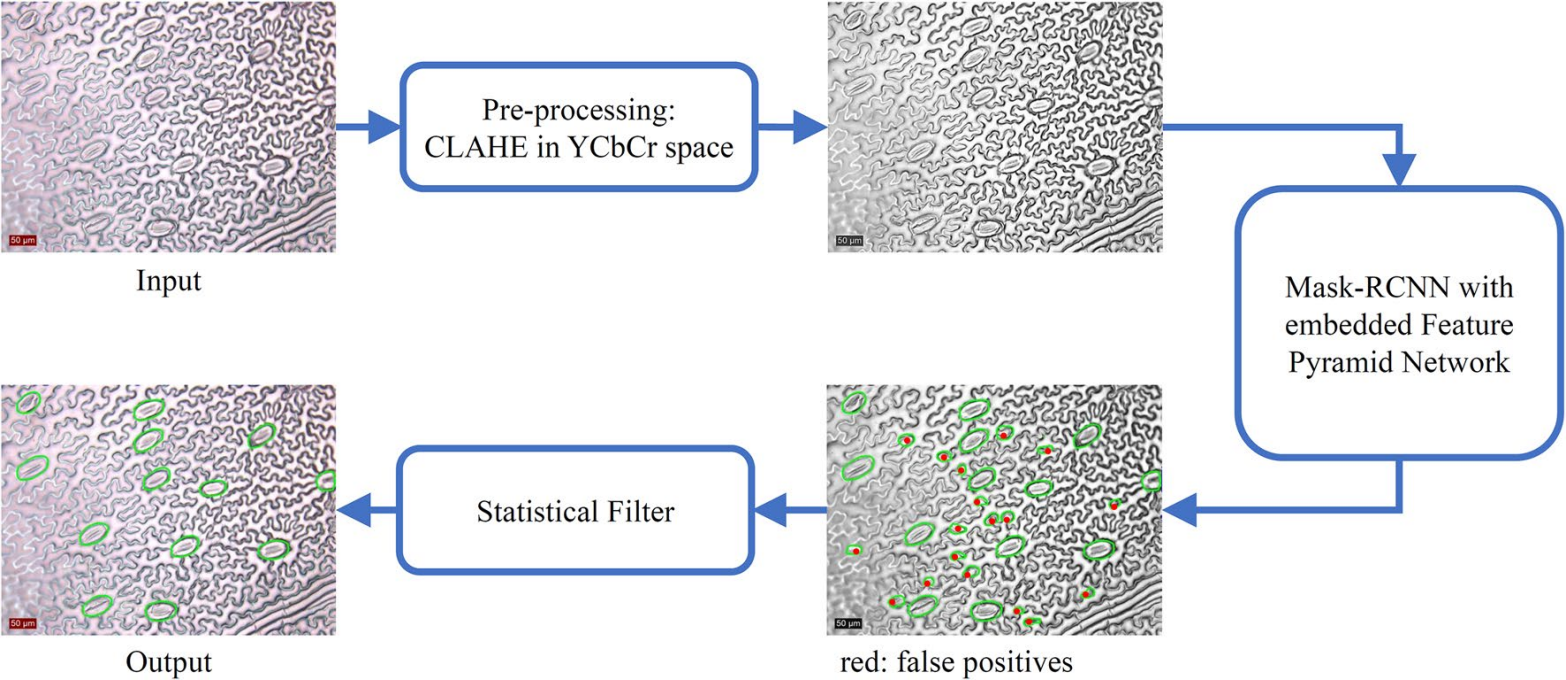
Proposed stomata detection pipeline. Image pre-processing removes colour space biases. Mask R-CNN instance segmentation detects stomata boundaries at different scales. Statistical filtering reduces the number of false positives generated by Mask R-CNN

The main aim of this research is to develop a generalised stomata detection and measurement platform which can robustly carry out instance segmentation across different microscope datasets. Input data can be collected from different plant species using a range of sample preparation and imaging techniques. The proposed methodology provides the end-user with the number of stomata on a given image, along with the area and the boundary coordinates for each individual stoma in the image. In short, the stomatal detection pipeline presented in this paper consists of three main stages as shown in Fig. 2. The first stage processes the microscope images such that biases introduced by different data preparation and imaging techniques are removed from the input dataset. The second and main stage of the pipeline, the Mask R-CNN algorithm, ensures stomata instances are detected across different scales. The final stage which consists of a statistical filter, removes false positives from the Mask R-CNN output, increasing the precision of the proposed solution. The solution is developed using Python3 backed by the OpenCV, Tensorflow and Keras libraries [39–41].

### Data preparation

Twelve microscope image datasets from 6 different sources were used for this research. The datasets cover a wide variety of plant types. A summary of the sample preparation methods, imaging methods and the image quality is provided in Table 1. The images quality categories, “high”, “medium” and “low”, were defined based on the following criteria.

- High: Image is sharp and detailed. Stomata are clearly visible.
- Medium: Image is somewhat blurred and has average colour contrast. However, stomata can be identified with respect to the background.
- Low: Image is mostly blurry. Artifacts such as dust, air bubbles and veins are present. Hard to discern stomata with respect to background elements.

Samples from 10 Gymnosperm species were collected at the Utrecht University botanical gardens. Samples were first macerated to the point that cuticle could be separated, following Lammertsma et al. [42]. After staining with Safranin, cuticles were mounted on glass slides in glycerol gelatine and imaged using a Leica^®^ Quantimet 500C optical microscope at $400 \times$ and $100\times$ zoom levels. Leaves of *Betula nana* specimen were obtained from three populations grown under sub-ambient, ambient and elevated CO$_{2}$ levels in growth chambers of the Utrecht Fytotron [43]. The sample preparation mechanism is similar to Gymnosperm datasets. Samples were imaged using an Olympus^®^ BH-2 optical microscope using $200\times$ and $400\times$ magnification levels.

The Ferns and Grass samples were collected at botanical gardens of Utrecht University and Amsterdam, Netherlands. Thirteen fern and 10 grass families are included in the dataset. Samples were collected by applying nail polish on leaf epidermis and lifting the print using clear Scotch tape. The samples were put through a 5% chlorine bleach before being mounted on microscope slides. The samples were imaged using a Leica^®^ DM6000-B microscope.

The UNSW-2019 dataset consists of samples from *Vitis vinifera*, *Prunus armeniaca*, *Citrus sinensis* and *Vinca major*. The samples were collected by applying nail polish on the leaf surface and lifting the imprint using clear tape. The tape was then mounted on a glass slide and imaged using an Aperio^®^ XT Brightfield Slide Scanner at 40$\times$ zoom.

The Eucalyptus dataset containing 27 species was sourced from publications by Schulze et al. and de Boer et al. [44, 45]. The samples were imaged using an Olympus^®^ BX51 optical microscope. The Poplar and USNM/USBG datasets were sourced from Fetter et al.’s publication on stomata detection [36]. The Cuticle and Ginkgo datasets were sourced from cuticle work carried out by Barclay et al. [12, 46]. The Google Images dataset consists of image search results for search query “stomata microscope”.

Altogether 3065 microscope images containing over 60,000 stomata was used for training and testing purposes. During the algorithm testing phase, some of these datasets were sub-divided based on image quality, which resulted in 16 datasets (see Table 3).

**Table 1:** Details of the datasets used for this research

Dataset	Quality	Preparation method	Imaging method	Source
Gymnosperm 400×	Med–High	Macerate and stain	Optical	This paper
Gymnosperm 100×	Low–High	Macerate and stain	Optical	This paper
Poplar	High	Nail polish	DIC	Fetter et al. [36]
Cuticle	Low–Med.	Clear and stain	Brightfield	Barclay et al. [12]
Ginkgo	High	Lamina peel	SEM	Barclay and Wing[46]
USNM/USBG	Low–Med.	Nail polish	DIC,SEM	Fetter et al. [36]
Betula Nana	Low–Med.	Macerate and stain	Fluorescence	This paper
Eucalyptus	Medium	Macerate and stain	Fluorescence	This paper
Ferns	Low–Med.	Nail Polish, 5% Cl bleach	Brightfield	This paper
Grass	Medium	Nail Polish, 5% Cl bleach	Brightfield	This paper
UNSW-2019	Med.–High	Nail polish	Brightfield	This paper
Google Images	Medium	Unknown	Unknown	Google

DIC: Differential Interference Contrast; SEM: Scanning Electron Microscope

**Fig. 3:**
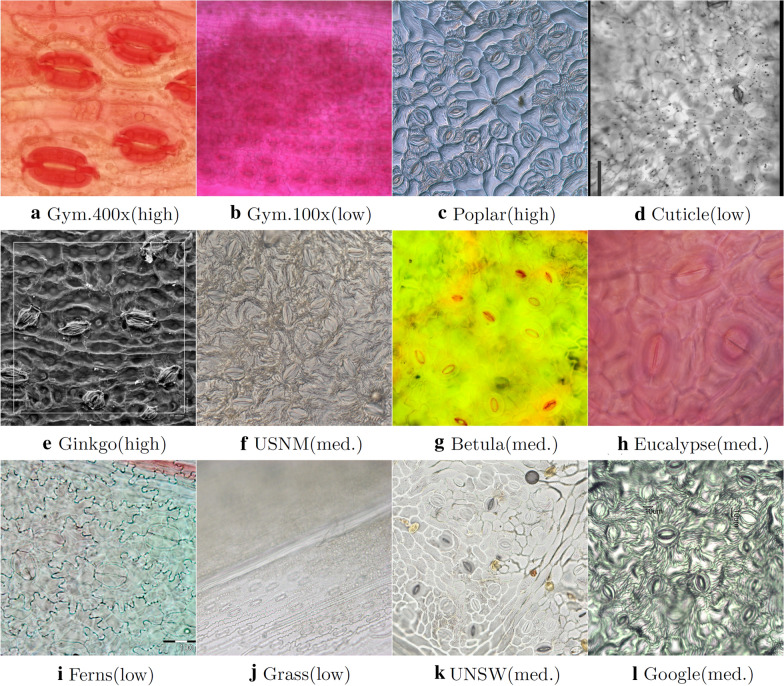
Images representing each dataset. Dataset name and quality provided in each sub-figure

### Stage 1: Image pre-processing

The quality and the colour of microscope images vary significantly depending on the sample collection and imaging techniques. The main aim of the image pre-processing step is to remove any colour space biases in the input image. This ensures that the final Mask R-CNN model is robust to images having unbalanced colour spaces (for example, high red-shift or high yellow-shift as shown in Fig. 3b and g). Contrast Limited Adaptive Histogram Equalisation (CLAHE), a common histogram equalisation method, is applied to the input image before being handed over to the Mask R-CNN model [47]. The exact steps of the process are as follows.

1. Convert the 3-channel colour image from RGB space to YCbCr space.
2. Apply Contrast Limited Adaptive Histogram Equalisation (CLAHE) on Y-channel.
3. Convert the image from YCbCr to RGB space.
4. Convert the image from RGB space to Grayscale and store it as a 3-channel .jpeg image.

The resulting image is fed into the Mask R-CNN which expects a 3-channel array as the input. The pre-processing steps assist Mask R-CNN to train without any colour space biases. Fig. 2 shows how the pre-processing step affects the image colour space.

### Stage 2: Mask R-CNN Algorithm

The proposed solution utilizes the Mask R-CNN algorithm to detect stomata. Compared to object detection algorithms such as SSD, YOLO, RCNN, Fast-RCNN and Faster-RCNN, which produce a single bounding box around the object of interest, Mask R-CNN focuses on instance segmentation, where the true boundary of the object is identified regardless of its shape [33, 35, 37, 48–50]. Thus, Mask R-CNN allows us to directly measure stomata orientation, axes lengths and overall area without any additional image processing steps. Such additional information further simplifies the process of developing algorithms which aim to measure finer characteristics of stomata, such as the pore opening. In addition to instance segmentation, Mask R-CNN leverages the capabilities of the Feature Pyramid Network (FPN) concept which uses the pyramidal nature of CNNs to detect objects at different scales [38]. Hence, Mask R-CNN is able to detect stomata of different sizes without having any prior knowledge on the magnification level of the input microscope image.

The Mask R-CNN implementation by Matterport is used as the base model for this work [51]. For this application, a Mask R-CNN model pre-trained on the MS-COCO dataset was employed to carry out transfer learning. 157 microscope images across 6 datasets were manually labelled to train the Mask R-CNN model. The manual stomata labelling process was carried out using the VGG Image Annotator (VIA) tool [52]. The aim was to train a general enough model using the minimum amount of training data by leveraging the capabilities of transfer learning. More information on the training data is provided in Table 2.

Several changes were made to the default Mask-RCNN training settings. An additional step of image augmentation was introduced to randomly rotate 2/3 of the input images, to ensure that the trained model is robust to stomata orientation. The anchor box scales for each feature pyramid level were set to [12, 24, 48, 96, 192] pixels in order to detect stomata of different sizes. The object detection confidence threshold was set to 50%. A batch size of 2 and a learning rate of 0.002 was adopted based on available computing resources. The model was trained for 85 epochs, where the input image was scaled such that the largest dimension is set to 1024 pixels. The model generated at epoch 51 was selected for testing purposes, to avoid any overfitting to training data. Additional details regarding the training setup can be found at https://github.com/Smart-Robotic-Viticulture/MaskStomata.

**Table 2:** Image data used for Mask R-CNN training and validation

Dataset	Training images	As a % of dataset	Val. images	Avg. stomata/image
Gymnosperm 40×	43	15.14%	14	6
Gymnosperm 10×	25	4.99%	16	27
Cuticle	28	4.20%	19	32
Ginkgo	15	7.61%	10	10
USNM/USBG	31	4.44%	30	26
Poplar	15	8.50%	10	33
Total	157	−	99	-

### Stage 3: Statistical filter

The Mask R-CNN algorithm outputs stomata detections along with corresponding boundary masks. In leaf epidermis microscopy, the stomata size does not vary much within an individual microscope image as the magnification is fixed and actual stomatal sizes are relatively uniform within a single leaf. However, the magnification level (and the resulting stomata size) can vary across a single dataset. In addition, the natural size of stomata varies between species. Nonetheless, the FPN embedded in Mask R-CNN, of which the main task is to detect objects at different scales, attempts to detect stomata at different scales for any given input image. This could result in false positives, especially when the image quality is low and other stomata-like structures are present (air bubbles, leaf structure) in the image. Therefore, a statistical filter is introduced to determine the actual scale of the stomata in a given image. The filter utilizes the prediction confidence values and the corresponding object areas to determine the appropriate stomata size range for a given image. Once the suitable stomata area range is calculated, all predictions outside this range are rejected. The following steps are implemented in the statistical filter:

1. Using Mask R-CNN output, select detections where the detection confidence is above the $90^{\mathrm{th}}$ percentile. Let’s call this collection {A}.
2. Calculate the average stomata area value for {A}.
3. From {A}, select the items where the stomata area is smaller than the average stomata area. Call this collection {$\hbox {A}_{s}$}.
4. From {A}, select the items where the stomata area is larger than the average stomata area. Call this collection {$\hbox {A}_{l}$}.
5. If there are more items in {$\hbox {A}_{l}$} compared to {$\hbox {A}_{s}$}, this indicates that the image contains “large” stomata. Now calculate the optimal stomata area (area_optimal) for that image by taking the average area value for {$\hbox {A}_{l}$}.
6. If there are more items in {$\hbox {A}_{s}$} compared to {$\hbox {A}_{l}$}, this indicates that the image contains “small” stomata. However, there could be a lot of “small” stomata detected due to noise in the image. Hence compare the average detection confidence score of {$\hbox {A}_{s}$} and {$\hbox {A}_{l}$}. If the detection confidence score is still higher in {$\hbox {A}_{s}$}, we can safely conclude that the image contains “small” stomata. Now calculate the optimal stomata area (area_optimal) for that image by taking the average area value for {$\hbox {A}_{s}$}.
7. From the original detections, select all stomata where the stomata area is, 0.65 $\times$ area_optimal < stomata area $\le$ 1.5 $\times$ area_optimal, despite their detection confidence value. Call this stomata collection {$\hbox {A}_{f}$}.
8. {$\hbox {A}_{f}$} includes all detections selected by the statistical filter. {$\hbox {A}_{f}$} is the final output of the algorithm.

The pseudo-code for the statistical filter is presented in Algorithm 1.

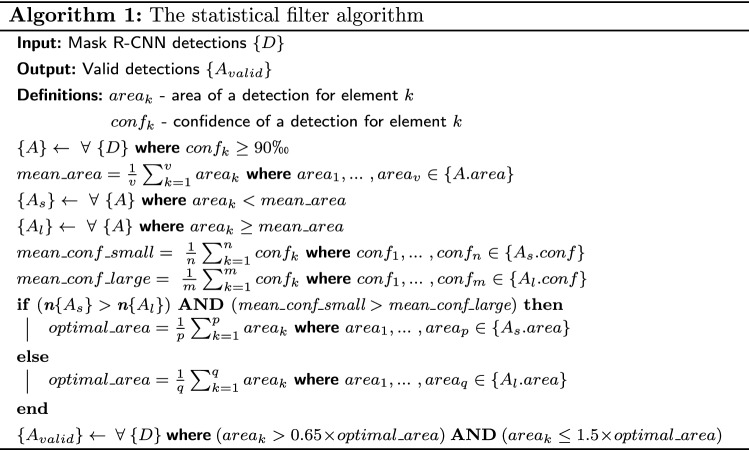



The coefficient values of 0.65 and 1.5 were chosen empirically to represent a 50% variation from the $optimal\_area$ value (i.e.: $0.65\times 1.5 \approx 1.0$ and $1.0\times 1.5 = 1.5$). The same coefficients were used across every experiment presented in this paper.

## Results

A series of experiments were conducted to evaluate the performance of the proposed Mask R-CNN based stomata identification system. The inference tasks were carried out on an Ubuntu 16.04 operating system, with 60 GB memory and an NVIDIA Tesla T4 GPU hosted on the Google Cloud Platform (GCP). On average, the algorithm takes 734ms to completely process an image. All input images were resized such that their width is set to 1024 pixels. The complete code for the project can be accessed at: https://github.com/Smart-Robotic-Viticulture/MaskStomata.

### Stomata detection performance

**Fig. 4:**
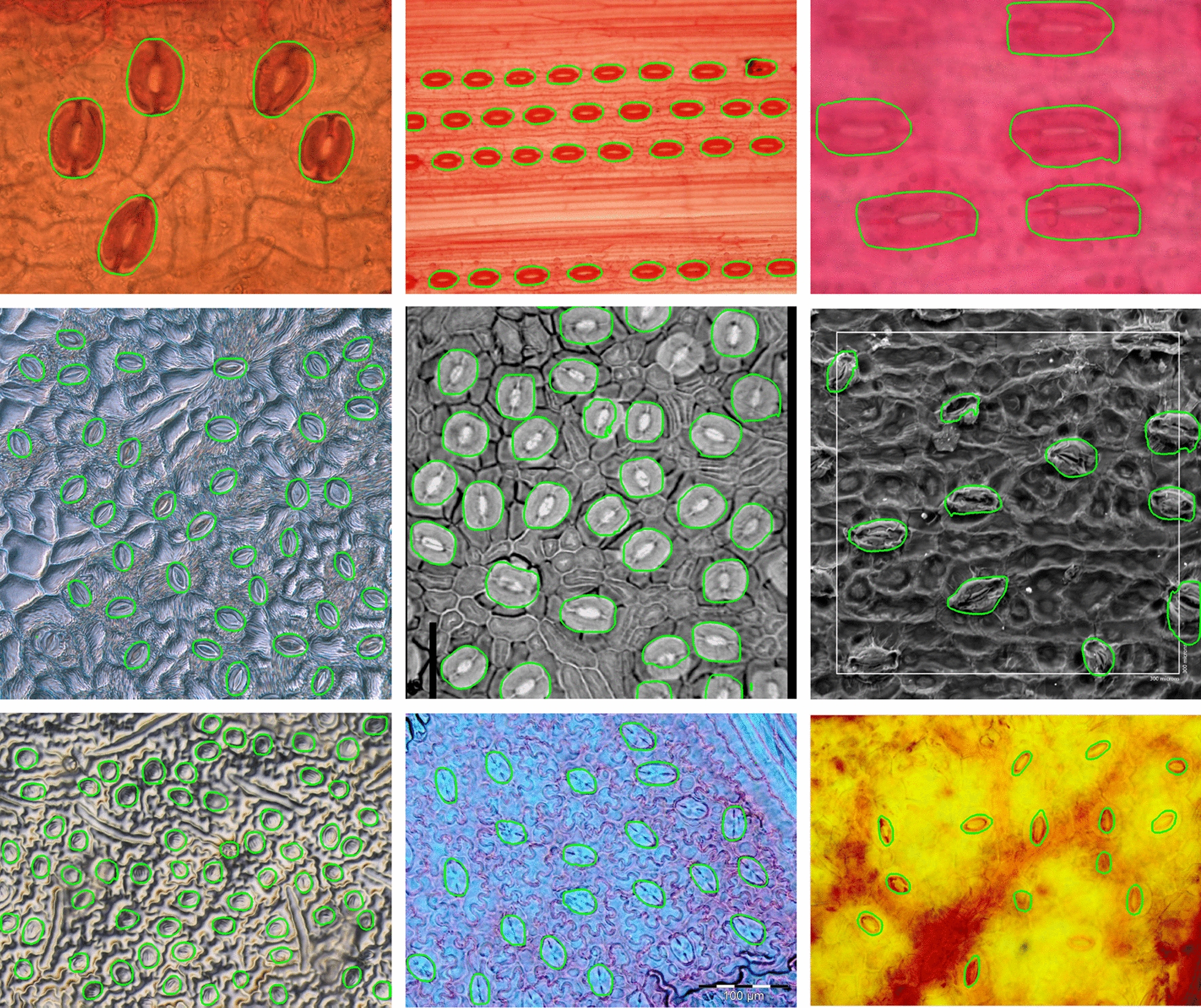
Some results for the proposed methodology. The method works well with stomata of difference sizes and quality

The stomata detection performance of the proposed methodology was evaluated against 12 different microscope datasets. Some datasets were separated into new sub-groups based on image quality as discussed in the previous section, resulting in 16 evaluation datasets. Figure 4 provides some examples which show the robustness of the proposed methodology against the image quality and stomata size. The final results are presented in Table 3. Out of the 16 datasets, the first 4 datasets are well known by the system, where data used for training sufficiently captures the stomata characteristics in these datasets. The average precision, recall, and F-score across these four datasets are 98.42%, 93.80%, and 96.05% respectively, despite the variation in image quality. The next 5 datasets in Table 3 contain images partially known to the system. This means a small percentage of images from these datasets were used for training, but they do not represent all plant types contained in those datasets. Hence, for partially known datasets, the trained neural network contains information on the sample collection and imaging procedure, but lacks plant leaf epidermis information for each individual species within the dataset. The average precision, recall and F-score for these 5 datasets are 94.60%, 79.76% and 86.48% respectively.

The last seven datasets in Table 3 include mostly low to medium quality data never seen by the neural network. Thus, the results provide important insight to the generalised nature of the proposed solution. This is the first time a stomata detection algorithm was tested against this many datasets unknown to the network. Apart from two low quality datasets, the proposed algorithm is able to produce 80+% F-scores for datasets unknown to the network, which shows the robustness of the proposed stomata detection pipeline. The precision values for the unknown datasets vary between 97.52% and 78.91% with the lowest precision value produced for the low quality ferns dataset (Ferns: low). Compared to previous tests, the recall values on average were 70.09% for data unknown to the neural network. Datasets with low image quality exhibit lower recall values, suggesting that the algorithm may be rejecting some stomata detections due to low confidence in the prediction due to quality.

**Fig. 5:**
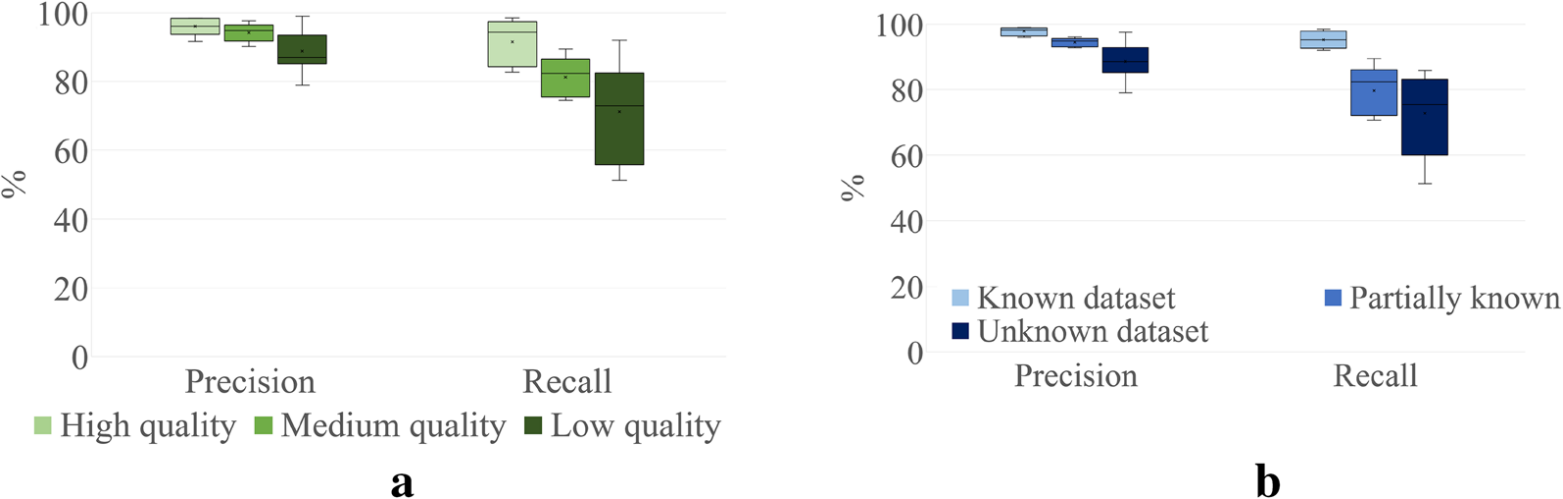
Stomata detection performance variance **a** based on image quality. **b** based on how well the dataset is known to the Mask R-CNN model. In both cases, recall drops while precision value is maintained

Results in Fig. 5a indicate that the algorithm maintains the precision of the detections despite the varying image quality. There is a performance drop in the recall value, as the algorithm has less confidence in its predictions (thus, rejecting them) as the image quality goes down. Figure 5b show how the detection performance varies based on how much a dataset is known to the model. Again, the conservative nature of the statistical filter ensures that the average precision variation is maintained within 10% across all datasets. However, the recall values show a higher variation with the average value dropping nearly 22% when dealing with unknown datasets.

The results presented in Table 3 also suggest that the proposed algorithm performance is more sensitive to image quality compared to other factors such as sample collection mechanism and stomata scale. The reduction of precision and recall is especially visible for low and medium quality datasets such as Ferns: low, Grass, Betula nana and Cuticle: low.

**Table 3:** Performance of the proposed stomata detection algorithm

Dataset	Quality	Known to model	Num. of stomata	Precision (%)	Recall (%)	F-Score (%)
Gymnosperm 400×	Med–High	Yes	944	95.87	98.41	97.12
Gymnosperm 100×: low	Low	Yes	10597	98.89	91.92	95.28
Gymnosperm 100×: high	High	Yes	7713	98.15	94.30	96.18
Poplar	High	Yes	5042	98.34	96.11	97.22
Cuticle: low	Low	Partially	8181	93.46	73.51	82.29
Cuticle: med	Medium	Partially	2631	94.80	89.43	92.04
Ginkgo	High	Partially	2802	96.02	82.65	88.84
USNM/USBG: low	Low	Partially	2569	92.70	70.65	80.19
USNM/USBG: med	Medium	Partially	16083	95.20	82.31	88.30
Betula nana	Low–Med	No	683	85.62	75.25	80.06
Eucalyptus	Medium	No	1088	93.22	83.46	88.07
Ferns: low	Low	No	964	78.91	51.24	62.14
Ferns: med	Medium	No	713	90.15	74.47	81.56
Grass	Low–Med	No	3288	85.20	55.66	67.32
UNSW-2019	Med–High	No	2242	91.53	85.77	88.56
Google Images	Medium	No	1496	97.52	76.34	85.64

### Stomata instance segmentation performance

**Fig. 6:**
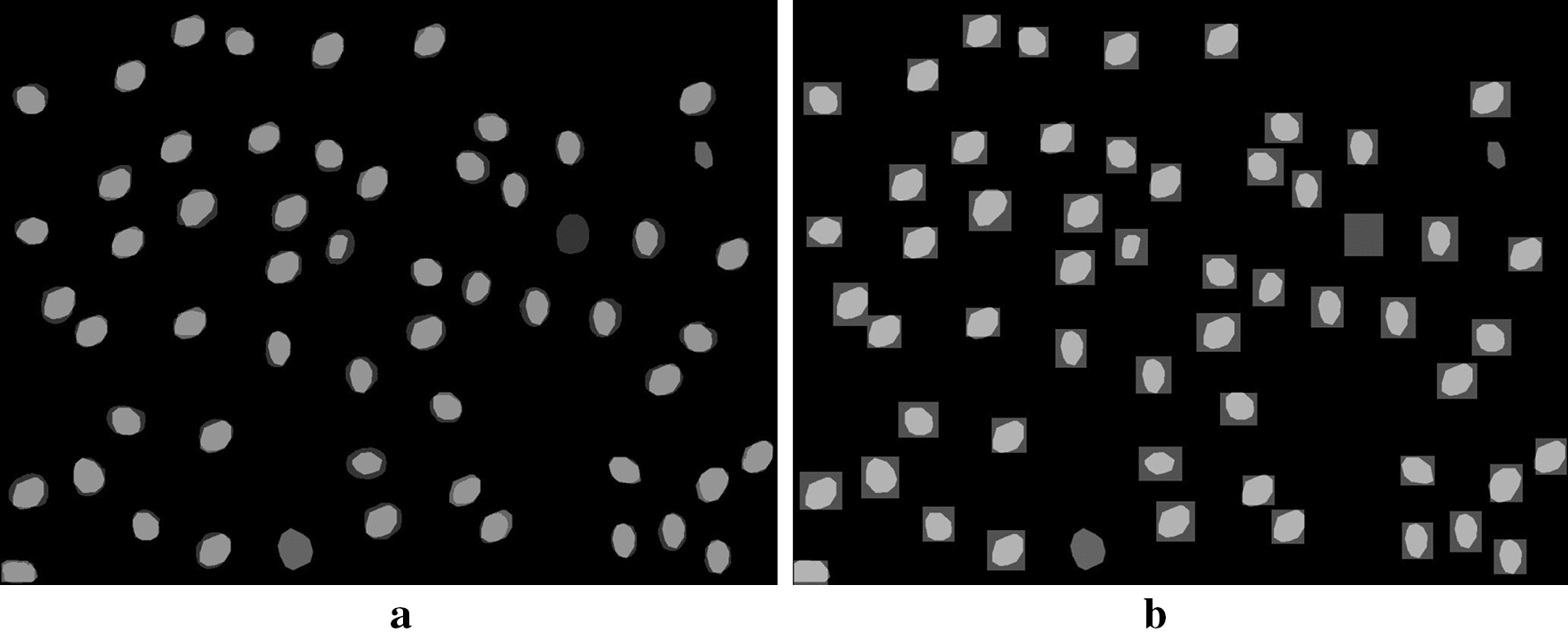
**a** Mask R-CNN overlap with ground-truth. **b** Bounding-box overlap with ground-truth. Lighter areas show overlap between ground-truth and the estimated output. Note: darker areas in both images represent false positives and false negatives

A key feature of the proposed algorithm is its ability to estimate the stomata boundary, thus providing an accurate result for the stomata area. In order to test the performance of this feature, the proposed algorithm was applied to 79 images where the stomata boundaries were manually marked to provide ground truths. These 79 images were sourced from datasets partially and fully known to the model. The IoU between the ground-truths and the stomata estimations were measured. Results were compared with that of a bounding box approach utilizing the same neural network architecture. Table 4 summarises the results. The proposed method easily outperforms the bounding box approach by 7% for IoU. Note that the results include errors due to false positives and false negatives. Figure 6 provides a visual example of how instance segmentation is far closer to the ground truth compared to a bounding box approach.

**Table 4:** Instance segmentation performance of the proposed method

Num. of images	Num. of stomata	Proposed method	Bounding box
		IoU	IoU
79	2386	0.70	0.63

Results are compared with a similar network structure which generates bounding boxes instead of instance polygons

### Effect of the statistical filter

As discussed in "Methods" section, we introduced a statistical filter to the stomata detection pipeline to improve the final output. The results presented in Table 3 include the statistical filter. Nevertheless, it is important to analyse how the statistical filter affects the final output of the system; thus, tests were conducted repeating the stomata detection process without the aid of the statistical filter. The corresponding unfiltered results for two datasets are shown in Table 5. The results show that the statistical filter clearly improves the precision of the algorithm. In both cases, the precision increases by around 10 % with the filter. However, the recall value drops by a similar percentage during this process. This is expected, as the algorithm is now being “more careful” before confirming stomata detections. In a practical sense, higher precision outweighs recall when there is a lot of data available. With higher precision, users can arrive at more accurate results relating to microscope images. Nonetheless, if needed, the user can sacrifice precision for recall by bypassing the statistical filter in the system.

**Table 5:** Effect of the statistical filter on stomata detection performance

Dataset	Quality	No filter (%)			With Statistical filter (%)		
		Precision	Recall	F-Score	Precision	Recall	F-Score
Ginkgo	High	88.15	92.00	90.00	96.02	82.66	88.83
Eucalyptus	Medium	83.17	90.24	86.56	93.23	83.46	88.07

The statistical filter improves the precision of the algorithm while sacrificing recall

### Fine-tuning the algorithm for a New Dataset

Results so far show that the proposed methodology performs well with different types of microscope datasets, including datasets never before seen by the neural network. However, there may be cases where the researchers would require higher accuracy and recall values than the ones produced by the proposed solution. For example, a researcher might have a limited number of low quality, microscope images where it is important to extract as much information as possible from available data. Results in Table 3 suggest that low quality images can have an impact on the performance, especially on recall. Hence, for such scenarios, we have provided a set of instructions on fine-tuning our stomata detector model with transfer learning. The relevant instructions can be found here: https://github.com/Smart-Robotic-Viticulture/MaskStomata. Table 6 presents how the model performance improves with transfer learning. For the low-quality Ferns dataset, we marked the ground truth on 12 training images containing around 14 stomata per image, using the VGG Image Annotator tool [52]. The labelling process took approximately 28 min. Then the Mask R-CNN was retrained for 40 epochs, with our existing stomata model providing the initial training weights. The fine-tuned algorithm drastically improves both precision and recall. Similar improvements were also found for the low-quality Grass dataset where 15 new training images (with labelling taking approximately 48 min) were introduced to the system.

**Table 6:** Performance improvement after fine tuning the stomata model to a specific dataset

Dataset	Quality	Train images	New precision (%)	New recall (%)	New F-score (%)
Ferns: low	Low	12	87.98 (+9.07)	81.13 (+29.89)	84.42 (+22.10)
Grass	Low–Med	15	90.91 (+5.71)	82.68 (+27.02)	86.60 (+19.28)

## Discussion

Results presented in the previous section indicate that the proposed Mask R-CNN based methodology performs well across different datasets containing stomata of varying size and quality. The test conducted using 7 microscope datasets previously not seen by the CNN model further solidifies the generalisable nature of the approach.

The results also suggest that image quality is the main factor affecting the performance of the solution. In addition to the reduction in the recall value, boundary estimation performance may also decrease in low quality images. However, more training images from low quality datasets could easily improve the performance of the system. Table 6 provides a couple of good examples supporting this case.

All input images were resized such that the image width is set to 1024 pixels. The authors expect this methodology to perform at its best when the original input image size is in this range.

## Conclusions

This paper presented a fully automated, high-throughput stomata instance segmentation methodology for microscope images. The proposed methodology combined a statistical filter with an FPN backed Mask R-CNN algorithm to accurately estimate the stomata boundary of a wide-variety of plant types. The algorithm was thoroughly tested against different datasets collected using different sample collection and imaging techniques. For the first time in this domain, the algorithm also tested against 7 datasets containing features never experienced by the network. Results show that the proposed method has an overall stomata detection precision, recall and F-score of 95.01%, 83.34% and 88.61% respectively, in a test conducted using over 2800 images containing over 60,000 stomata.

The next step would be to use these results to accurately measure other morphological traits of the stomata such as pore dimensions and guard cell widths. The authors also intend further improve the model presented in this paper by adding more training samples from additional plant types.

## Data Availability

The datasets used and/or analysed during the current study are available from the corresponding author on reasonable request. The complete code for the project can be accessed at: https://github.com/Smart-Robotic-Viticulture/MaskStomata.
